# Potential influence of parental copy number variations on noninvasive prenatal testing (NIPT): two case reports

**DOI:** 10.1186/s13039-020-00485-3

**Published:** 2020-05-25

**Authors:** Yiming Qi, Jiexia Yang, Yaping Hou, Rong Hu, Dongmei Wang, Haishan Peng, Aihua Yin

**Affiliations:** 1 Prenatal Diagnosis Centre, Guangdong Women and Children Hospital, Guangzhou, 511400 Guangdong China; 2 Maternal and Children Metabolic-Genetic Key Laboratory, Guangdong Women and Children Hospital, Guangzhou, 511400 Guangdong China

## Abstract

**Background:**

Small subchromosomal deletions and duplications caused by copy number variants (CNVs) can now be detected with noninvasive prenatal testing (NIPT) technology. However, the clinical utility and validity of this screening for CNVs are still unknown. Here, we discuss some special conditions in which both cases simultaneously exhibited false positives caused by maternal CNVs and false negatives due to limitations of the technology.

**Case presentation:**

In case 1, NIPT indicated a 1.1 Mb deletion at 21q21.1, but the umbilical cord for array CGH (aCGH) revealed a 422 kb deletion at 15q13.3. Peripheral blood of the parents for aCGH showed a 1.1 Mb deletion at 21q21.1 in the mother’s sample, and the same deletion at 15q13.3 was detected in the father’s blood. In case 2, NIPT showed a 1.5 Mb deletion at 22q11.21, but aCGH of amniocytes revealed a 1.377 Mb duplication rather than a 1.5 Mb deletion at 22q11.21. Furthermore, aCGH analysis of the parental blood revealed a 647 kb deletion at 22q11.21 in the mother and a 2.8 Mb duplication of 22q11.21 in the father.

**Conclusions:**

Our findings not only highlight the significance of diagnostic testing following a positive cfDNA sequencing result but also the necessity for additional analytical and clinical validation before routine use in practice.

## Background

Noninvasive prenatal testing (NIPT) by massively parallel sequencing of cell-free fetal DNA (cffDNA) fragments has already become a mainstream technology in conventional aneuploidy screening since it was introduced in 2011. The high accuracy in detecting fetal trisomies 21, 18 and 13 has been widely verified in numerous studies, with both a sensitivity and a specificity of > 99% [1–3]. NIPT for common chromosomal aneuploidies is used increasingly in clinical practical advances [4–7]. More recently, some companies and laboratories have expanded NIPT to cover a number of microduplication/microdeletion syndromes caused by copy number variants (CNVs) [8], and Chen et al. [9] and Liang et al. [10] demonstrated that NIPT performed well in microduplication/microdeletion syndromes.

Microduplication/microdeletion syndromes (MMSs) caused by CNVs are relatively rare, accounting for 1–2% [11] of all newborn congenital abnormalities, but they often result in a severe burden for both families and society. The most common microdeletion, 22q11.2 deletion syndrome (known as DiGeorge syndrome (DS)) [12], is even more common than T18 and T13 combined [13, 14]. Thus, early detection of these subchromosomal imbalances is important, as it will help identify high-risk pregnancies and offer the possibility of a confirmatory invasive diagnostic test. However, there is still much to know about the clinical utility and validity of this screening for CNVs, and most of these clinically relevant CNVs occur in pregnancies lacking ultrasound anomalies. Therefore, it is often an accidental discovery of NIPT.

Here, we provided some special conditions of maternal CNV false positives and false negatives using low coverage massively parallel sequencing. In these two cases, the cfDNA result was false positive because of maternal rearrangements and false negative because of NIPT limitation. Thus, NIPT is a screening test. These results not only highlight the significance of diagnostic testing following a positive cfDNA sequencing result but also the necessity for additional analytical and clinical validation before routine use in practice.

## Materials and methods

### NIPT

Whole blood samples of 5 to 10 mL from pregnant women were collected in EDTA tubes within 8 h. Afterwards, JingXin Fetal Chromosome Aneuploidy (T21, T18, and T13) Testing Kits (CFDA registration permit No. 0153400300) were used to perform cfDNA extraction, library construction, quality control, and pooling. Then, a semiconductor sequencer, the JingXin BioelectronSeq 4000 System (CFDA registration permit NO. 20153400309), was used for DNA sequencing. Sequencing reads were filtered and aligned to the human reference genome (hg19) [1]. Fetal and maternal chromosome copy number variations (CNVs) were classified with our modified Stouffer’s z-score method as described previously [15]. Here, each chromosome with an absolute value of the z-score greater than 3 was marked with chromosome aneuploidies or microdeletions/microduplications [15].

### Array CGH

Umbilical cord blood samples or amniotic fluid were collected from the fetuses. Peripheral blood (5 mL) was collected from each parent. Genomic DNA was extracted from the fetal samples and peripheral blood using the QIAamp Genomic DNA Blood Mini Kits (Qiagen, Hilden, Germany). The DNA samples were heated by fragmentation, labeled, and hybridized to the Agilent 4X44k microarray (Agilent Technologies, Inc., Santa Clara, CA, USA) according to the standard protocol. The data were analyzed by Agilent CytoGenomics software, version 2.7.22.0.

## Case presentation

### Case 1

Patient 1 was a 28-year-old, gravida 1 para 0 woman whose elective triple marker screen revealed a low risk of trisomy 21/18/13. Nuchal translucency (NT) was 1.3 mm at 13 gestational weeks. However, with the help of prenatal ultrasound series monitoring, a mild echogenic bowel (grade 1) and hydramnios with amniotic fluid volume (AFV) 74 mm were noted at 28 gestational weeks. The patient’s past medical history was significant only for Southeast Asian deletion α^0^-thalassemia (−-^SEA^). Two months prior to pregnancy, she started taking 0.4 mg folic acid and calcium tablets. Furthermore, the social history of the patient was negative for alcohol, tobacco, and illicit drug use before/during pregnancy.

**Fig. 1:**
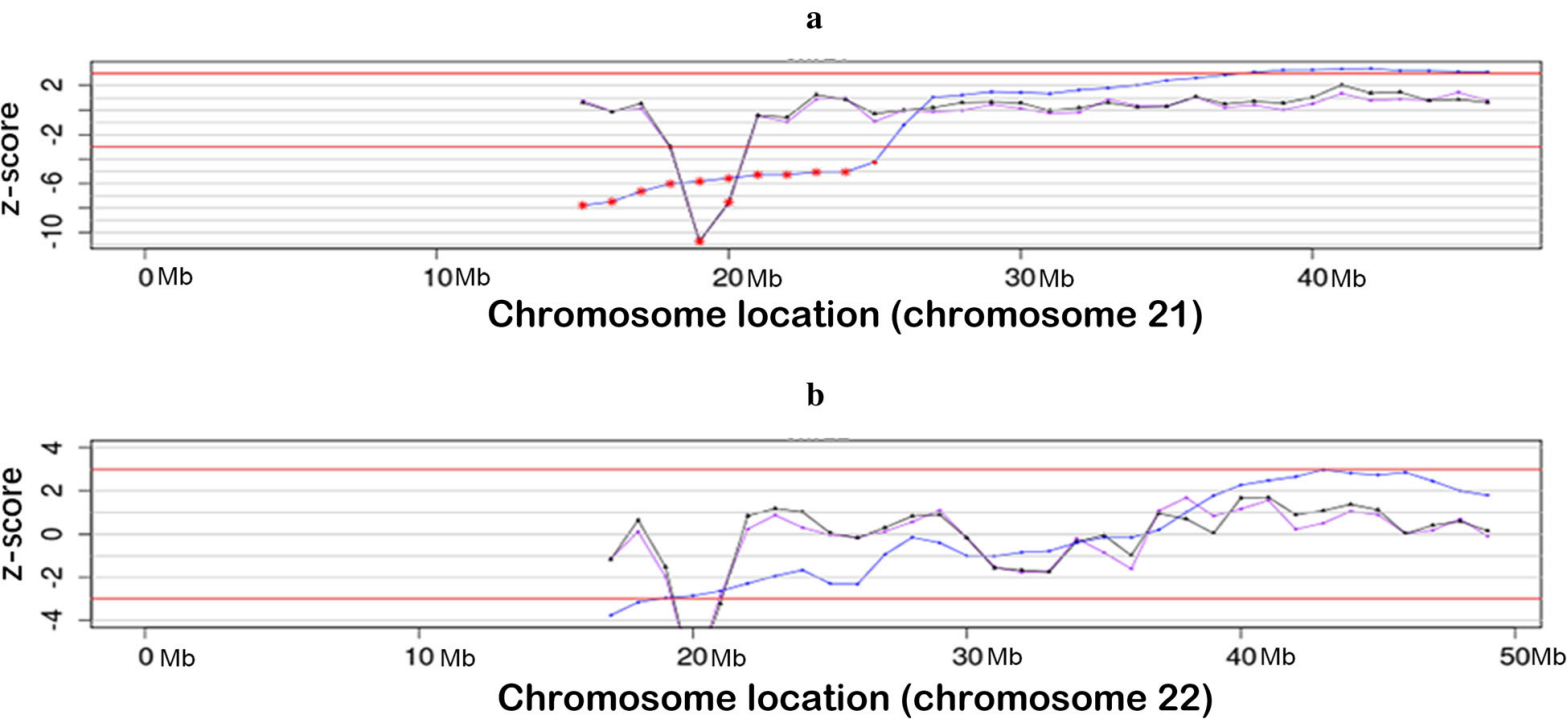
NIPT results of the two cases. **a**, case 1; **b**, case 2. The blue line is the Stuffer z-score. The purple line and black line are the corrected z-score. Vertical axis: z-score. Horizontal axis: Chromosome localization

**Fig. 2:**
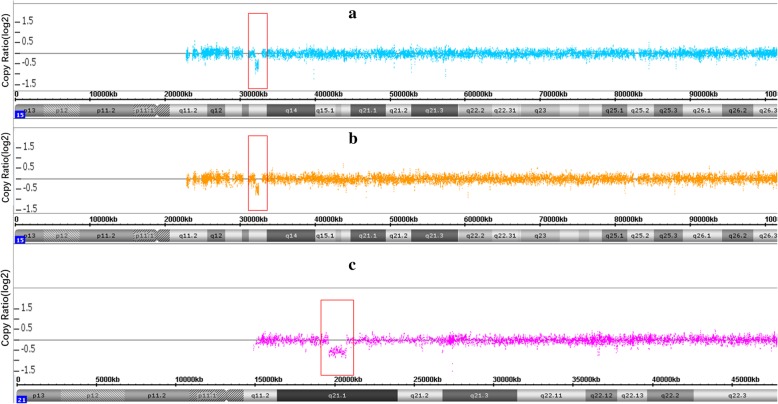
Array-CGH results of case 1. **a**, fetal; **b**, paternal; **c**, maternal. Vertical axis: Copy Ratio (log2). Horizontal axis: Chromosome localization

NIPT based on low coverage (0.1×) whole-genome sequencing using an Ion Proton Sequencer (CapitalBio Technology Inc., Beijing) was performed at 28 gestational weeks, and it suggested that there was a 1.1 Mb deletion, with a z-score of − 10.76, located at 21q21.1. The estimated fetal DNA concentration in the maternal plasma was 19.7% on analysis (Table 1 and Fig. 1a). To explore the precise details, umbilical cord blood samples were punctured at 29 gestational weeks for the aCGH analysis. However, the aCGH analysis revealed a 422 kb deletion at 15q13.3, rather than the 21q21.1 deletion (Fig. 2a).

**Table 1:** Summary of test results of NIPT and aCGH in this two cases

Case	NIPT				CMA		
	cffDNA	CNV	z-score	Reads	sample of fetus	Peripheral blood of mother	Peripheral blood of father
Case 1	19.70%	1.1 Mb deletion at 21q21.1	-10.76	4.29 Mb	arr[hg19] 15q13.3(32021609-32444043)×1	arr[hg19] 21q21.1(19731098-20834451)×1	arr[hg19] 15q13.3(32021609-32444043)×1
Case 2	14.70%	1.5 Mb deletion at 22q11.21	-6.71	5.93 Mb	arr[hg19] 22q11.21(18935464-20312661)×3	arr[hg19] 22q11.21(20716876-21363447)×1	arr[hg19] 22q11.21(18636749-21461017)×3

After extensive counseling, the patient told us that her husband had a history of epilepsy, which made us reconsidered this result. We then collected the peripheral blood of the parents for aCGH analysis. The mother’s blood showed a 1.1 Mb deletion at 21q21.1. The father’s blood showed the same deletion (422 kb deletion) at the region of 15q13.3 (Fig. 2b and c).

As is routine practice, an ultrasound was conducted to monitor the developmental status of the fetus, and no obvious abnormalities were recorded. The parents decided to continue the pregnancy. A male baby 3160 g and 51 cm high was delivered at term with no phenotypic abnormalities, and the Apgar score was 9 at 5 min. As follow-up, the neonate was normal in growth and psychomotor development at 6 months of age.

### Case 2

**Fig. 3:**
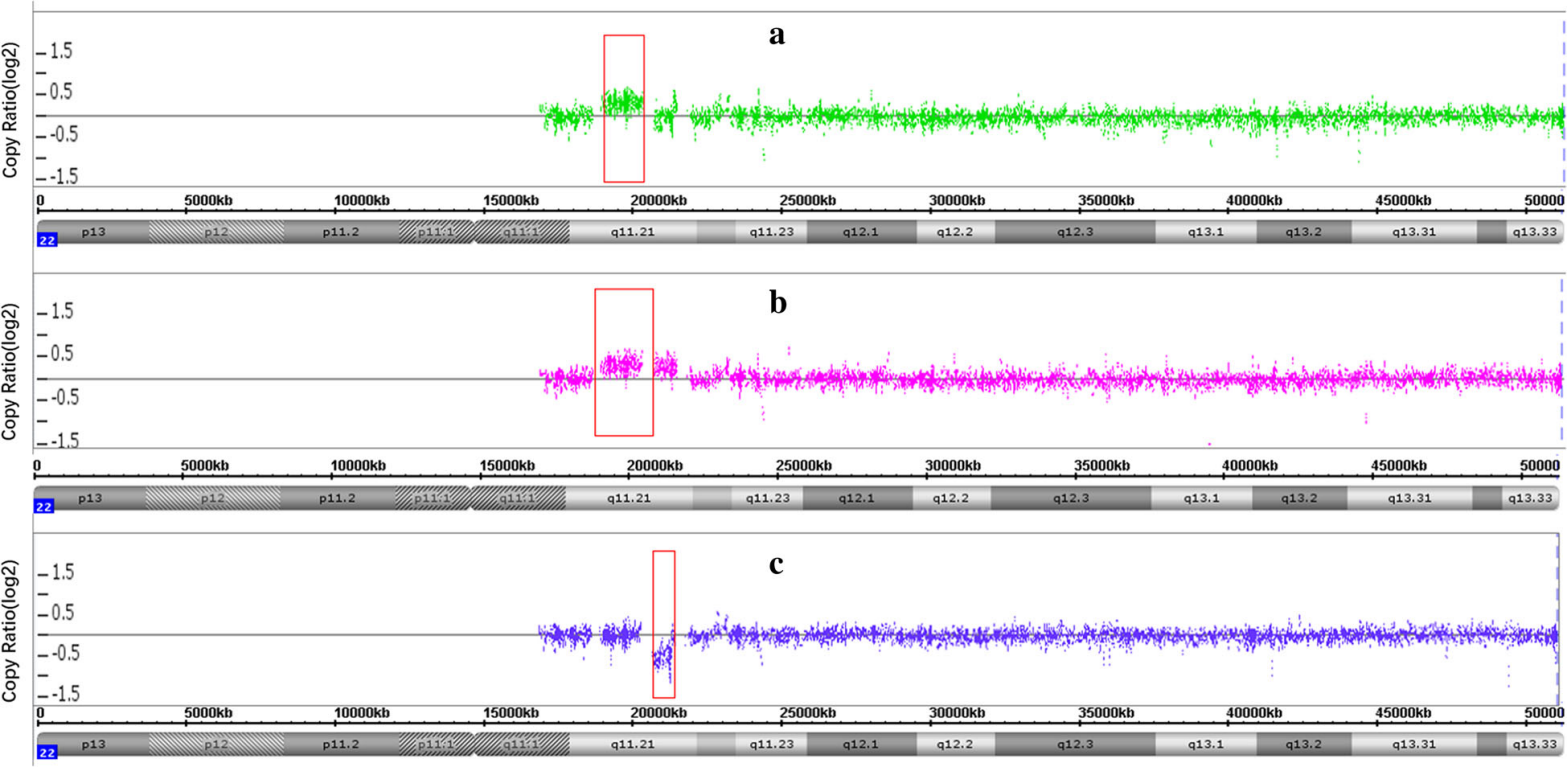
Array-CGH results of case 2. **a**, fetal; **b**, paternal; **c**, maternal. Vertical axis: Copy Ratio (log2). Horizontal axis: Chromosome localization

A 31-year-old, gravida 6 para 1 woman presented for prenatal counseling at 12 gestational weeks. First-trimester serum screening (FTS) indicated that the fetus was at intermediate risk of trisomy 21 syndrome (1/750) in another hospital. The woman had experienced three spontaneous abortions and one labor induction for intestinal malformation. She asked for NIPT at 13 gestational weeks due to FTS results and abnormal reproductive history. NIPT revealed a 1.5 Mb deletion at 22q11.21, which overlaps the critical region of DiGeorge syndromes. The cffDNA was 14.7%, and the z-score was − 6.71 (Table 1 and Fig. 1b). Conventional cytogenetic analysis of cultured amniocytes revealed a normal karyotype of 46, XY in all 40 cultured colonies. Then, aCGH was applied to the remaining uncultured amniocytes, and aCGH revealed a 1.377 Mb duplication rather than a 1.5 Mb deletion at 22q (Fig. 3a). The result of the fetus was arr[hg19] 22q11.21(1935464–20312661)×3. Furthermore, aCGH analysis of the parental blood samples was performed to explore the real origin of the CNVs. It revealed the result of arr[hg19] 22q11.21(20716876–21363447)×1, indicating a 647 kb deletion of 22q11.21 in the mother, and an arr[hg19] 22q11.21(18636749–21461017)×3, indicating a 2.8 Mb duplication of 22q11.21 in the father (Fig. 3b and c); the area almost overlapped with the CNV of the fetus. Karyotypes were interpreted using the International System for Human Cytogenetic Nomenclature 2016 (ISCN 2016) criteria [16]. Continuous prenatal ultrasound monitoring and III level ultrasound screening at 24 gestational weeks did not find remarkable malformations. The location and volume of the fetal thymus and fetal echocardiogram were normal. A male infant with a weight of 2870 g and height of 46 cm was born at 37 + ^3^ weeks with no phenotypic abnormalities, and the Apgar score was 8 at 5 min.

## Discussion and conclusions

Aneuploidy screening using cell-free DNA (cfDNA) has recently been expanded to include selected microdeletions. More recently, some companies and laboratories have expanded NIPT to detect MMSs caused by CNVs [8–10]. Expanding NIPT to include detection of specific conditions caused by a CNV [17] (such as 22q11.2 [18], 1p36 [19], cri-du-chat (5p15.3) [20], Prader-Willi (paternal 15q11-q13) [21] and Angelman (maternal 15q11-q13) [22]) is technically possible. However, validation has been limited, the sensitivity and specificity of detection of CNVs by NIPT are still unknown, and there are some special conditions where maternal CNV causes inconsistent results. In the present study, both cases were very special. False positives caused by maternal CNVs and false negatives simultaneously exist due to limitations of the technology. Thus, large sample clinical studies are still needed for its validation.

From 2015 to 2019, a total of 44,423 pregnant women underwent NIPT tests in our prenatal diagnostic center, with a total false positive number of 66 and a total false negative number of 2. NIPT used in this study was performed by a semiconductor sequencing platform (SSP). A previous study reported that the overall sensitivity and specificity of this platform for detecting trisomy 21, 18 and 13 combined were 99.61 and 99.91%, respectively [1]. In addition, a recent study reported that NIPT performed well in detecting subchromosomal microdeletions/microduplications with a large clinical sample size through this platform [8]. Moreover, we are exploring the detection of other chromosomal abnormalities using this platform [23]. However, NIPT is a screening test, and all the abnormal results underscore the need for additional validation before routine use in practice as well as the necessity for confirmatory diagnostic testing after a positive cfDNA result.

The detection power of NIPT is determined mostly by fetal fraction, CNV size, and sequencing depth. Liao et al. [24] reported that at a mean fetal fraction of 14%, for CNVs< 5 Mb, the detection rate was only 14.3%. For case 1, the real fetal CNV sizes were 422 kb. Even though the cffDNA was 19.7%, exceeded 14%, and the sequencing depth was 4.29 Mb, the chances of successful detection of CNVs of this size still approached zero. The results further demonstrated that CNV size is one of the most important factors influencing the detection rate of NIPT detecting chromosome microduplication/microdeletion.

In case 1, the father suffered from infantile-onset epilepsies 1 year after birth, although no obvious organic lesions, such as cortical malformations, tuberous sclerosis, and perinatal brain injury, were detected by brain magnetic resonance imaging (MRI). Congenital cardiac defects were not observed in the father, which might be attributed to the low penetrance of the 15q13.3 microdeletion for major structural abnormalities. As mentioned above, it is reasonable that the risk of epilepsy in the fetus is relatively high.

CNVs are an important source of normal and pathogenic variants. In case 2, the fetus had a 1.377 Mb duplication, the father had a 2.8 Mb duplication on 22q11.21. The main clinical features of 22q11.2 duplication syndrome include hypocalcemia arising from parathyroid hypoplasia, thymic hypoplasia, and outflow tract defects of the heart (OMIM: 188400). The mother had a 647 kb deletion on 22q11.21. However, neither the mother nor the father seemed to have any abnormal clinical phenotype. Therefore, the parents decided to continue pregnancy. The CNV data in case 2 showed the amplification region on chr. 22 of the fetus, which was smaller than that in the father. One of the possibilities was that the fetus carried one chr. 22 from the father with the amplification and the second chr.22 with deletion from the mother. This meant that the possibility that the fetus had two CNVs on chr. 22 was high.

Positive detection results predicted by NIPT should be followed by diagnostic testing to confirm the fetal origin, as well as by parental studies to establish inheritance and to provide accurate counseling for patients.

## Data Availability

The datasets used and/or analyzed during the current study are available from the corresponding author on reasonable request.

## Abbreviations

NIPT: Non-invasive prenatal testing
CNV: Copy-number variants
aCGH: Array-CGH
DS: DiGeorge Syndrome
FTS: First-trimester serum screening
cfDNA: cell free DNA
NT: Nuchal Translucency
MMSs: Microduplication/microdeletion syndromes
SSP: Semiconductor sequencing platform
MRI: Magnetic Resonance Imaging
AFV: Amniotic fluid volume
--^SEA^: Southeast Asian deletion α^0^-thalassemia
ISCN: International System for Human Cytogenomic Nomenclature
